# Proton beam radiation therapy for vestibular schwannomas-tumor control and hearing preservation rates: a systematic review and meta-analysis

**DOI:** 10.1007/s10143-023-02060-x

**Published:** 2023-07-04

**Authors:** Antonio Santacroce, Mioara- Florentina Trandafirescu, Marc Levivier, David Peters, Christoph Fürweger, Iuliana Toma-Dasu, Mercy George, Roy Thomas Daniel, Raphael Maire, Makoto Nakamura, Mohamed Faouzi, Luis Schiappacasse, Alexandru Dasu, Constantin Tuleasca

**Affiliations:** 1 European Radiosurgery Centre Munich, Munich, Germany; 2 Department of Medicine, Faculty of Health, Witten/Herdecke University, Witten, Germany; 3 Department of Neurosurgery, St. Barbara-Klinik Hamm-Heessen, Hamm, 59073 Germany; 4 University of Medicine and Pharmacy “Gr. T. Popa”, Iasi, Germany; 5 Neurosurgery Service and Gamma Knife Center, Centre Hospitalier Universitaire Vaudois, Lausanne University Hospital (CHUV), Rue du Bugnon 44-46, BH-08, CH-1011 Lausanne, Switzerland; 6 Faculty of Biology and Medicine (FBM), University of Lausanne (UNIL), Lausanne, Switzerland; 7 Oncology Pathology Department, Karolinska Institutet and Stockholm University, Stockholm, Sweden; 8 Medical Radiation Physics, Stockholm University, Stockholm, Sweden; 9 ENT Department, Lausanne University Hospital (CHUV), Lausanne, Switzerland; 10 Department of Neurosurgery, Academic Hospital Köln-Merheim, Köln, 51058 Germany; 11 Department of Medicine, Faculty of Health, Witten/Herdecke University, Witten, 58455 Germany; 12 Division of Biostatistics, Center for Primary Care and Public Health (Unisanté), University of Lausanne, Lausanne, Switzerland; 13 Radiation Oncology Department, Lausanne University Hospital (CHUV), Lausanne, Switzerland; 14 The Skandion Clinic and Uppsala University, Uppsala, Sweden; 15 Medical Radiation Sciences, Department of Immunology, Genetics and Pathology, Uppsala University, Uppsala, Sweden; 16 Ecole Polytechnique Fédérale de Lausanne (EPFL, LTS-5), Lausanne, Switzerland

**Keywords:** Proton therapy, Vestibular schwannoma, Radiosurgery, Facial nerve, Hearing

## Abstract

**Objective:**

Proton beam therapy is considered, by some authors, as having the advantage of delivering dose distributions more conformal to target compared with stereotactic radiosurgery (SRS). Here, we performed a systematic review and meta-analysis of proton beam for VSs, evaluating tumor control and cranial nerve preservation rates, particularly with regard to facial and hearing preservation.

**Methods:**

We reviewed, using the Preferred Reporting Items for Systematic Reviews and Meta-Analyses (PRISMA) articles published between 1968 and September 30, 2022. We retained 8 studies reporting 587 patients.

**Results:**

Overall rate of tumor control (both stability and decrease in volume) was 95.4% (range 93.5–97.2%, *p* heterogeneity= 0.77, *p*<0.001). Overall rate of tumor progression was 4.6% (range 2.8–6.5%, *p* heterogeneity < 0.77, *p*<0.001). Overall rate of trigeminal nerve preservation (absence of numbness) was 95.6% (range 93.5–97.7%, *I*^2^ = 11.44%, *p* heterogeneity= 0.34, *p*<0.001). Overall rate of facial nerve preservation was 93.7% (range 89.6–97.7%, *I*^2^ = 76.27%, *p* heterogeneity<0.001, *p*<0.001). Overall rate of hearing preservation was 40.6% (range 29.4–51.8%, *I*^2^ = 43.36%, *p* heterogeneity= 0.1, *p*<0.001).

**Conclusion:**

Proton beam therapy for VSs achieves high tumor control rates, as high as 95.4%. Facial rate preservation overall rates are 93%, which is lower compared to the most SRS series. Compared with most currently reported SRS techniques, proton beam radiation therapy for VSs does not offer an advantage for facial and hearing preservation compared to most of the currently reported SRS series.

## Introduction

Vestibular schwannomas (VS) arise from the vestibular branch of the eight cranial nerve and account for approximately 8% of the intracranial neoplasms [1]. The most common symptoms are hearing loss, tinnitus, and balance disturbance.

Therapeutic management options for VSs include observation, microsurgical resection, and radiation therapy [2]. Large VSs with symptomatic mass effect have a strong indication for microsurgical resection [3]. Stereotactic radiosurgery (SRS) has a long-term clinical and scientific track record for small to medium size VSs [4].

Proton therapy has been suggested as a way to diminish side effects by reducing the radiation dose to tissues at risk [5]. While photon radiation delivers its maximum dose almost immediately upon entry into tissue, protons have a finite range in tissue and deliver most of their dose at the end of their range. This is known as the Bragg peak phenomenon, and a sharp dose falloff occurs just beyond it [6]. Thus, one might assume that fractionated proton beam therapy (FPRT) may be associated with better hearing preservation because of the unique dosimetric properties of proton radiotherapy, with rapid dose fall off distally and laterally to the irradiated target.

Here, we perform a systematic review and meta-analysis of the role of proton beam radiation for VSs. Our primary aim was the evaluation of tumor control. Our secondary aim was the assessment of cranial nerve preservation, particularly in relationship to hearing. The purpose of the present systematic review and meta-analysis is to recapitulate the current literature specific to proton therapy for VS.

## Methods

### Systematic review and meta-analysis

A systematic review of the literature was performed using the Preferred Reporting Items for Systematic Reviews and Meta-Analyses (PRISMA) approach [7].

### Eligibility criteria

We included both retrospective and prospective studies, written in English, which have reported patients with VSs treated by proton beam therapy, independently of history of previous surgery or not.

We excluded studies written in languages other than English.

### Information sources

Our information sources were Medline, Pubmed, Embase, Scopus, and Web of Science databases.

### Search strategy

We searched for articles published between 1968 and September 30, 2022.

The following MESH terms or combination of those were used either in title/abstract: “proton” AND “vestibular schwannoma(s)” (15 results) and “proton” AND “acoustic neuroma(s)” (11 results). Four independent reviewers (AS, MFT, AD, CT) have screened the content of all articles and abstracts (Table 1).

**Table 1:** Basic demographic and dosimetric data

	Type of study	Number	Follow-up	Age	Male:female	Prior surgery /NF2	Tumor volume	Dose/relative biological effectiveness (RBE)	Median radiation dose to 90% of the cochlea (D90)
Harsh et al. (2002)	Retrospective	68	Mean 44 (6–96)	Mean 67 (36–86)	36:32	9/68, -	Mena 2.49 mL (0.3–12.7)	12 Cobalt Gy equivalent at 70% isodose line	
Bush et al. (2002)	Retrospective	29	Mean 34 (7–98)	Mean 53 (21–80)	15:14	-, 3/29	4.3 mL	54.0 Cobalt Gy in 30 fractions If useful hearing: 60 Cobalt Gy in 30–33 fractions	
Weber et al. (2003)	Retrospective	88	Median 38.7 (12–102.6)	Median 69.2 (36.1–91.9)	46:42	15/88, -	1.4 mL (0.1–15.9)	Median 12 Cobalt Gy to 70–108% isodose line in 3 fractions (2–4)	
Vernimmen et al. (2009)	Retrospective	51	Median 71 (24–149)	Mean 50 (20–85)	23:28	Prior surgery: 14/51 NF2: 5/51		26 cobalt gray equivalent (cGyE) in 3 fractions	
Barnes et al. (2018)	Prospective	96 (94 assessed)	Median 64	Median 56 (21–60)	45:50	14/95, -	1.4 mL (0.7–3.7)	59.4 Gy (no serviceable hearing, n=43) 54 Gy (serviceable hearing, n=34) 50.4 (serviceable hearing, n=19)	
Zhu et al. (2018)	Retrospective	14	Median 68 (36–106)	60 (24–74)	8:6	0/14, -	6.4 mL (0.3–16)	50.4 Gy in 28 fractions	
Koetsier et al. (2021)	Retrospective	221 (136 single and 85 fractionated) Primary: 128/187 59/187	Median 54 (mean 59; 12–143)	64 (37–86)	1:1	34/221 187/221 primary	Single: 0.6 mL (0.3–1.3) Fractionated: 0.9 (0.5–2)	Single: 12 Gy Fractionated: subjective useful hearing, larger tumors, postoperative residual tumors	SRS: mean 8.9 (RBE), 1.8–12.1 FT: 44.2 (RBE), 27.7–53.9
Saraf et al. (2022)	Prospective single arm phase 2	20 (all serviceable hearing)	Median 48 (12–60)	Median 64	6:12	0/20	Median 0.81 mL (0.54–1.95)	50.4–54 in 28–30 fractions of 1.8 Gy (RBE) each	Median 40.6 Gy RBE versus 46.9 RBE (serviceable vs non serviceable hearing at 1 year) *p*=0.08

**Fig. 1:**
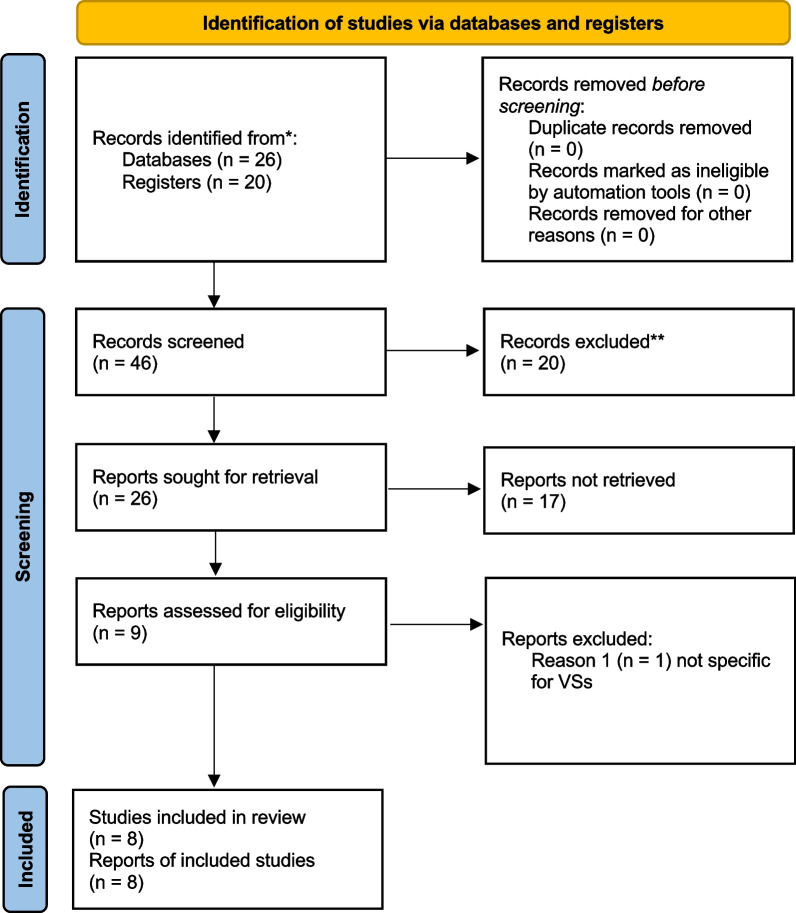
Prisma flow diagram

One article identified in the initial search strategy reported treatments of multiple types of schwannomas, without separately detailing the outcomes for each one of them and was further excluded [8]. Eight studies [6, 9–15] reporting 587 patients met the inclusion criteria. Two were prospective (single arm) [9, 12] while the others were retrospective. Figure 1 shows the flow diagram of the article selection process.

### Outcome measures

Primary outcome measure was tumor control. Secondary outcome measures were cranial nerve preservation, particularly for hearing and facial nerve. Development of hydrocephalus post-treatment requiring shunt placement was also noted.

### Statistical analysis using OpenMeta (Analyst)

Due to the high variation in study characteristics, a statistical analysis using a binary random-effects model (DerSimonian-Laird method) was performed. We used OpenMeta (Analyst) from the Agency for Healthcare Research and Quality.

Weighted summary rates were determined using meta-analytical models. Testing for heterogeneity was performed for each meta-analysis. Pooled estimates using meta-analytical techniques were obtained for all the individual outcomes previously described in the same section.

## Results

### Tumor control

**Fig. 2:**
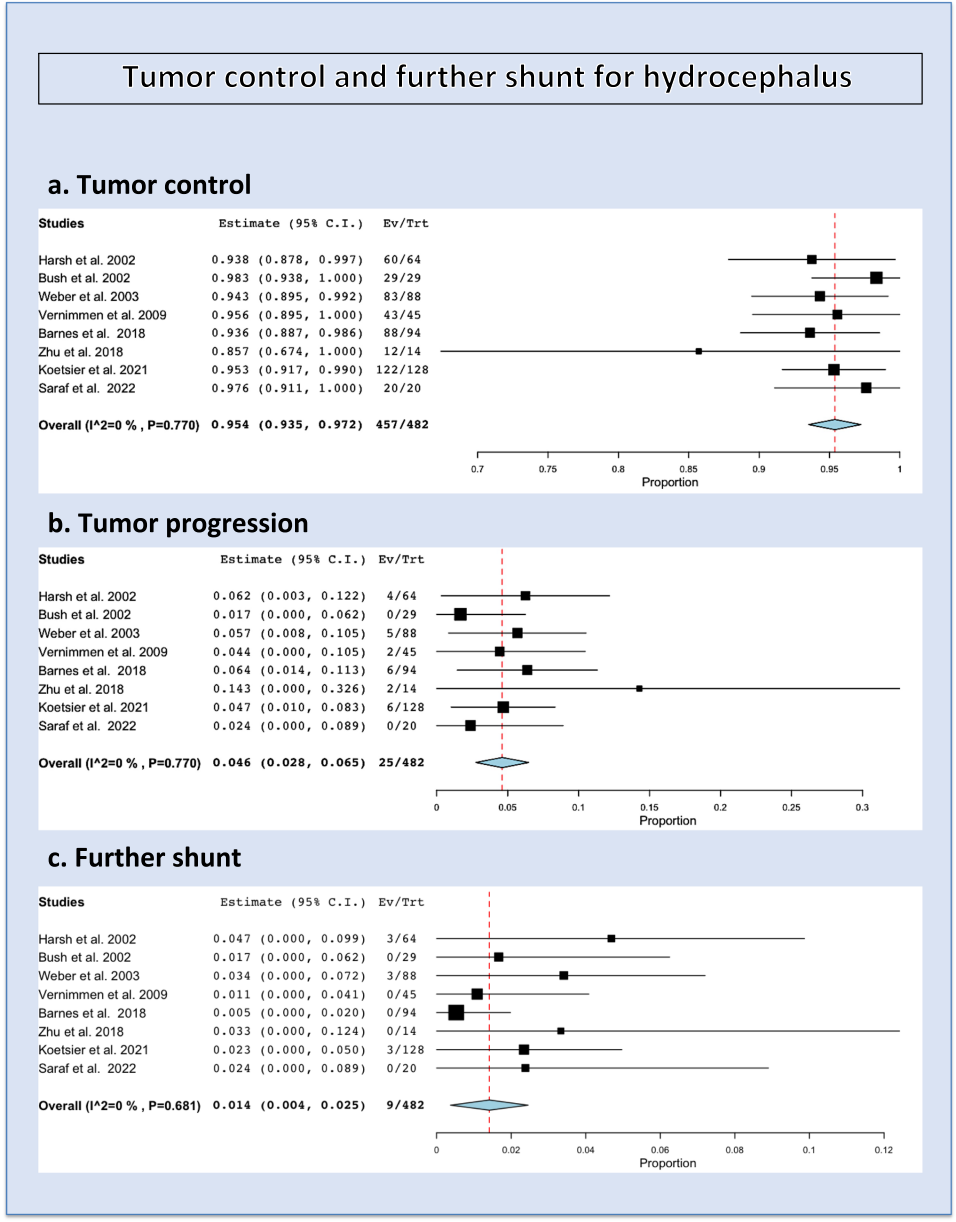
Tumor control (**a**), tumor progression (**b**), and further shunting (**c**)

The overall rate of tumor control (both stability and decrease in volume) was 95.4% (range 93.5–97.2%, *p* heterogeneity= 0.77, *p*<0.001; Fig. 2, a; Table 2).

The overall rate of tumor progression was 4.6% (range 2.8–6.5%, *p* heterogeneity < 0.77, *p*<0.001; Fig. 2, b).

### Hydrocephalus requiring shunt placement

The overall rate of hydrocephalus requiring shunt placement was 1.4% (range 0.4–2.5%, *p* heterogeneity= 0.68, *p*= 0.008; Fig. 2, c).

### Trigeminal nerve preservation

**Fig. 3:**
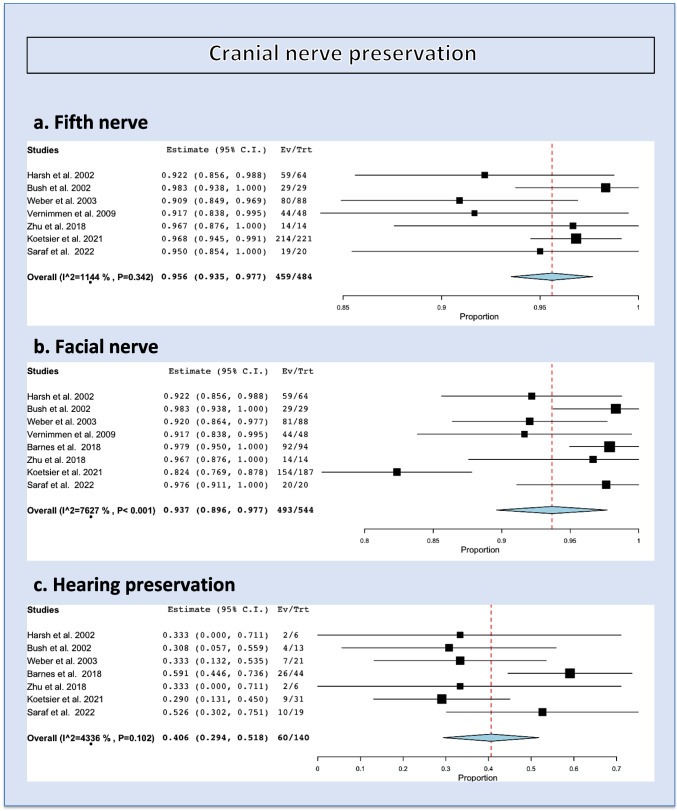
Cranial nerve outcome: trigeminal (**a**), facial (**b**), and hearing preservation (**c**)

The overall rate of trigeminal nerve preservation (absence of facial numbness) was 95.6% (range 93.5–97.7%, *I*^2^ = 11.44%, *p* heterogeneity= 0.34, *p*<0.001; Fig. 3, a; Table 2).

### Facial nerve preservation

The overall rate of facial nerve preservation was 93.7% (range 89.6–97.7%, *I*^2^ = 76.27%, *p* heterogeneity<0.001, *p*<0.001; Fig. 3, b; Table 2).

### Hearing preservation

The overall rate of hearing preservation was 40.6% (range 29.4–51.8%, *I*^2^ = 43.36%, *p* heterogeneity= 0.1, *p*<0.001; Fig. 3, c; Table 2).

## Discussion

The current systematic review and meta-analysis reports high rates of tumor control, as high as 95.4% (range 93.5–97.2). With regard to cranial nerve preservation, the overall rate of facial nerve preservation was 93.7% (range 89.6–97.7%) and the overall rate of trigeminal nerve preservation (absence of facial numbness) was 95.6% (range 93.5–97.7%). The overall rate of hearing preservation was disappointing, at 40.6% (range 29.4–51.8). Thus, proton beam therapy for VSs achieves high tumor control rates with modest rates of hearing preservation [6]. Moreover, the chances of facial nerve preservation are lower compared with most radiosurgery techniques.

**Table 2:** Outcomes after proton beam radiotherapy: tumor control, cranial nerve preservation rates

	Local control	Serviceable hearing preservation rates	Serviceable hearing preservation details	Cranial nerves ARE	Cranial nerve preservation	Shunt
Harsh et al. (2002)	2 y: 94% 5 y: 84% 60/64: controlled: • 35/64: regression • 25/64: unchanged 4/64: progression (1 salvage SRS)	2/6	2/6 preserved (1/6 improved 1/6 unchanged) 4/6 lost hearing	2/64 new facial weakness 5/64 new facial paresthesias	V: 59/64 VII: 59/64 VIII: 2/6	3/64
Bush et al. (2002)	29/29: controlled • 11/29 regression • 18/29: unchanged	-	4/13	2/29, vertigo/ataxia, all resolutive with a short course of corticosteroid therapy	V: 29/29 (100%) VII: 29/29 (100%)	-
Weber et al. (2003)	2 y: 95.3% 5 y: 93.6% 83/88: controlled 5/88: progression	-	7/21	-	At 5 y V: 89.4%; 8/88 permanent after a median time of 4.9 months (0.27–23.2) VII: 91.1%; transient in 4/88 after a median of 2 months (0.4–6.6); VII permanent: 7/88, grade II in 3, grade III in 3 and grade IV in 1 after a median time of 5.1 months (1.5–7.9)	3/88
Vernimmen et al. (2009)	2 y: 98% 5 y: 98% 10 y: 87% 43/45: controlled 2/45: progression	42%	2 y: 74% 5 y: 42% 10 y: 42%	2/48 severe facial palsy (grade IV)	V: 44/48 V: 2 y: 96%; 5 and 10 y: 93% VII: 44/48, 4/48 of which 2 mild (grades I&II) and 2 complete (grade IV) VII: 90.5% at 10 y	-
Barnes et al. (2018)	59.4 Gy: 95% 54 Gy: 97% 50.4 Gy: 92% (*p*= 0.8) 10 y: 90% 88/94: controlled 6/94: progression 4/94: further surgery	4y: 54 Gy: 44% 50.4 Gy (64%) (*p*= 0.2) Hearing decrease after 14.8 months (4.7–49)	7/16 (54 Gy) 19/28 (50.4 Gy)	Major CN toxicity: 2/94 VII: 2/94 facial paralysis V or VII: 7/94 transient facial and/or trigeminal nerve dysfunction 5%	V: - VII: 92/94	-
Zhu et al. (2018)	3 y: 85% 12/14: controlled 2/14 surgery (1 with 16 mL volume; 2 with 4.9 mL)	-	2/6	No V and no VII GR1: 2/14 (skin changes) GR2: 1/14 headache No acute GR3 toxicity	V: 14/14 VII: 14/14	-
Koetsier et al. (2021)	5 y: 96% (95% CI 90–98%) Controlled: 122/128 6/128: further treatment Single: 5/128 (2 re-irradiation, 3 surgery) RT: 1/59 (surgery)	1y: 42% (SDS loss)	9/31	V: trigeminal neuralgia -single: 3/128 -RT : 4/59 VII : HB II: -single: 5/128 -RT : 1/59 HB III–IV: -single: 2/128 -RT : 0/59 HB V–VI: -single: 0/128 -RT : 0/59 VIII: severe dizziness: -single: 7/128 -RT : 6/59	V: 214/221 VII: 154/187 • Single: 97/128 • Fractionated: 57/59	Single: 3/128 Fractionated: 0/59
Saraf et al. (2022)	4 y: 20/20 (100%)	1 y: 53% (95% CI 29–76%) Primary endpoint not yet reached	10/19 GR 1: 9/15 GR 2: 1/4 Both PTA and WRS worsened at 1 year (*p*<0.0001)	G1: 1/20: V-th dysfunction G2: 9/20 (7/20 hearing impairment) G3: 1/20 (hearing impairment)	V: 19/20 VII: 20/20	0/20

For proton beam therapy, indications for fractionation were [6] subjectively useful hearing, larger tumors, and postoperative residual tumors. Larger tumors showed shrinkage more often than smaller tumors [6]. Moreover, cystic tumors showed shrinkage in a higher proportion [6] (*p*= 0.08). In terms of local control, previous studies of tumor control rates after fractionated radiotherapy for VSs in large series show tumor control rates that range from 84 to 95% [16–19].

Overall rates of trigeminal nerve preservation are very good with proton beam therapy. Some risk factors for cranial nerve injury have been identified. Trigeminal neuralgia occurs more often in patients with larger tumors volumes (*p*=0.005) [6] and in patients who received fractionated RT (*p*= 0.05) [6].

Overall facial nerve preservation rates in the present study are approximately 93%, with is rather disappointing, as much lower compared to current SRS techniques, as reported by Tsao et al. [20] between 95 and 100%. With regard to the facial nerve, Weber et al. [14] suggested that facial neuropathy was associated with prescribed dose, maximal dose, as well as the inhomogeneity coefficient (dose inhomogeneity).

For hearing preservation, Barnes et al. [9] identified, based on multivariate analysis, that initial tumor diameter (<=1.5 cm) is a better prognostic factor for maintaining serviceable hearing (*p*= 0.01) after proton therapy. In the prospective series of Saraft et al. [12], D90 has a trend towards worse hearing outcomes (median 40.6 Gy [RBE] versus 46.9 Gy [RBE] (*p*= 0.08). The doses to the cochlea [12] were relatively higher compared with other fractionated RT studies, suggesting that the proton dose distribution might not help in sparing function of the cochlea. The European Particle Therapy Network recommends limiting the dose to the cochlea at 45 Gy in fractionated radiotherapy regimens, while other authors suggested even lower doses [21–23]. Previous dosimetric studies have shown a clear clinical impact of the dose received by the cochlea [24–28].

Some authors suggest that the primary mechanism of radiation damage of nonproliferating organs is through late-term fibrosis and vascular damage [12]. On the other hand, it has also been proposed that fractionated stereotactic radiotherapy (FSRT) delivered over a period of weeks may spare cochlear function, based on the assumption that there is a radiobiological recovery of normal tissue while using fractionated treatments. The serviceable hearing rates with such studies have ranged between 54 and 84% at 5 years [29–33]. The prospective study of Saraf et al. [12] concluded that fractionated proton beam radiation therapy for VS did not meet the goal of serviceable hearing preservation. The authors further suggested that the dose to the cochlea correlates with hearing preservation independent of treatment modality. Saraf et al. [12] did not find a statistically significant association between tumor size, tumor volume, total RT dose, age, and affected ear or baseline ipsilateral organs at risk (OAR) as related to serviceable hearing at 12 months. Bragg peak sparing seems to not be useful for VSs, when the cochlea is close to the tumor.

The recent systematic review and meta-analysis of the role of stereotactic radiosurgery for VSs by Tsao et al. [20] suggested that hearing preservation rates for single fraction SRS series are difficult to compare to other modalities due to the reduction of SRS prescribed physical dose over time [4]. In large series of patients treated with single fraction SRS, and doses between 12 and 14 Gy, the 5-year hearing preservation rate ranged from 41 to 79%. Four series reported no statistically significant difference between single fraction and fractionated stereotactic radiotherapy in terms of hearing preservation [17, 34–36]. Furthermore, treatment technique is another relevant consideration, as earlier studies were most likely to include patients treated with passive scattering, with more recent ones having used active scanning. There is a trend towards better hearing preservation in some newer reports (2018–2022) in comparison to the earlier ones (2002–2003), although this is not universal (as seen in the Fig. 3c).

Future studies should ideally focus on cochlea and vestibulum sparing dosimetry, evaluation of cognitive functioning, quality of life, and risk of secondary cancer, to further determine whether the higher costs of proton radiotherapy are justified for VSs patients [6]. Radiobiology [37, 38] and particularly biologically effective dose delivered to the tumor might play a role both in tumor control [39] and hearing preservation [40].

The main limitations, as individually suggested by the published studies are as follows: a wide range of follow-up duration, treatment technique, heterogenous manner of outcome reporting, dose selection, cochlear sparing or not, and tumor sizes (with several studies including small volumes).

## Conclusion

The present systematic review and meta-analysis suggests that proton beam therapy for VSs achieves high tumor control rates. However, the existing literature does on proton therapy for VS does not offer evidence for an advantage in hearing preservation compared to standard SRS techniques. Moreover, the chances of facial nerve preservation are lower compared with most radiosurgery techniques.

In sum, compared with most current radiosurgery techniques, proton beam radiation therapy for VSs does not offer an advantage for facial and hearing preservation compared to most of the currently reported stereotactic radiosurgery series.
